# Editorial: The Deadly Secrets of *C. difficile*—Insights Into Host-Pathogen Interaction

**DOI:** 10.3389/fmicb.2022.897612

**Published:** 2022-04-06

**Authors:** Meina Neumann-Schaal, Uwe Groß, Ingo Just, Dieter Jahn

**Affiliations:** ^1^Leibniz-Institute DSMZ–German Collection of Microorganisms and Cell Cultures, Braunschweig, Germany; ^2^Braunschweig Integrated Centre of Systems Biology (BRICS), Braunschweig, Germany; ^3^Institute of Medical Microbiology and Virology, University Medical Center Göttingen, Göttingen, Germany; ^4^Institute of Toxicology, Hannover Medical School, Hanover, Germany; ^5^Institute of Microbiology, Technische Universität Braunschweig, Braunschweig, Germany

**Keywords:** *Clostridioides* (*Clostridium*) *difficile*, toxins A and B *Clostridium difficile*, binary toxin CDT, *Clostridium difficile* infection (CDI), anaerobic metabolism

## Introduction

*Clostridioides* (formerly *Clostridium*) *difficile* is an anaerobic, spore-forming bacterium, widely distributed in soil, water, animals and the gut of healthy humans (Hall and O'Toole, 1935; al Saif and Brazier, 1996; Lawson et al., 2016). The symptoms of *C. difficile* infection (CDI) range from relatively mild diarrhea to severe life-threatening pseudomembranous colitis, toxic megacolon and a subsequent sepsis (Lessa et al., 2015). In the last two decades, an emerging number of nosocomial and community-acquired infections was reported worldwide (Bartlett, 2006; Rupnik et al., 2009; Lessa et al., 2015). CDI is commonly, but not necessarily associated with previous administration of antibiotics. Further risk factors are age, cancer treatment and immunosuppression. However, CDI also affects individuals without these classical risk factors (Bignardi, 1998; Rupnik et al., 2009).

According to the US Center for Disease Control and Prevention (CDC), *C. difficile* caused almost half a million infections in the USA each year, two-third of the infections were health-care associated^1^. About 1 in 6 patients suffers from recurrent infections. Within a month of diagnosis, 9% of the patients over age 65 died of a healthcare-associated CDI in the US in 2015 (Lessa et al., 2015). According to the European Center for Disease Prevention and Control (ECDC), the burden of healthcare-associated CDIs in acute care hospitals in the US was estimated at 300,000 cases in 2015 (Lessa et al., 2015).

## Methods

The challenges of genetic modifications, handling and cultivation procedures of *C. difficile* require individualized methods of analyzing *C. difficile* compared to other bacteria. Maikova et al. summarized recent advances in understanding the CRISPR-Cas system in *C. difficile* and discussed possible applications. Trautwein et al. developed a novel metabolic labeling strategy with ^15^N-labeled media for quantitative proteomics in *C. difficile*. Beyond the current ribotyping techniques, Emele et al. developed a proteotyping approach for the rapid identification ribotype 027 isolates.

## The Impact and Functionality of Toxins

*C. difficile* harbors various secreted and surface proteins responsible for colonic colonization and subsequent inflammatory reactions. The most important and best characterized ones are three large clostridial toxins: toxin A and toxin B and in some bacterial strains, the binary toxin CDT. The phage-derived holin system is involved in toxin export in *C. difficile*. Mehner-Breitfeld et al. report on the different TcdE isoforms present in *C. difficile* and their effect on cell growth and lysis. Following export, the toxins have a wide range of effects on the host cell system. Comparison of the Ras-glucosylating activity of the two toxins from *C. difficile* with the lethal toxin of *Clostridium sordellii* by Genth et al. showed that lethal toxin and toxin A glucosylate Ras toxin B does not. Toxin B but not toxin A is primarly responsible for inflammatory responses. Further articles of the Research Topic focus on role and functionality of toxin B. Junemann et al. focus on the quantitative phosphoproteome of epithelial cells in response to toxin B treatment. Their data show the response of more than 1,200 phosphosites, predominantly a response of the MAPK-dependent signaling pathways. The role of Cys-2232 of toxin B was evaluated by Chung et al.; its exchange indirectly reduced binding to two identified toxin B receptors, namely FZD2 and RVRL3. In a further report on toxin receptors, Schöttelndreier et al. report that the putative cell surface receptors for toxin B including ZD2/7, CSPG4, and PVRL3 are in fact not internalized after toxin binding and, thus are merely binding proteins. Toxin B is further an interesting target for drug development. Fühner et al. report on the development of neutralizing and non-neutralizing antibodies by targeting different epitopes of toxin B.

## Epidemiology and Evolution

Infections with *C. difficile* have been reported from many countries worldwide. However, there seems to be differences in clade and toxinotype distributions that also reflect different clinical outcomes. In humans, colonization by non-toxigenic *C. difficile* strains are associated with a lower risk of CDI. Dayananda and Wilcox review the effect of co-infecting or colonizing on the infection of *C. difficile* strains to identify potential exploitable mechanisms to prevent *C. difficile* infection. Hernández et al. studied the fecal microbiota associated with *C. difficile* infection and *Akkermansia* to be predictive for the presence of a *C. difficile* infection and highlight that co-infection with other pathogenic agents are to be considered in treatment. In three further publications, the focus is set on the worldwide impact of *C. difficile*. Li et al. report on an outbreak in an intensive care unit of a teaching hospital in China and highlight the importance of routine testing in a clinical setting. A comparative study by Seugendo et al. shows the different prevalence at sites in Ghana, Indonesia and Germany reflecting different ribo- and toxinotypes and resistances likely reflecting the distinct use of antibiotics. In a study with non-toxigenic isolates from patients in Mexico, Camorlinga et al. highlight heterogenous but still distinctive phenotypic characteristics that differentiate them from toxigenic strains.

## Physiology and Host Response

During gut colonization *C. difficile* is exposed to the host immune response, multiple stress factors and also bacteriophages. Fortier summarized the current knowledge on *C. difficile* bacteriophages and additionally discussed the role of phages in the therapeutic effectiveness of fecal microbiota transplantation and its therapeutic perspectives. The structure of the bacteriophage tail-like bacteriocin of *C. difficile* itself was studied by Schwemmlein et al. In a study by Bernal et al., they provided a first link between *C. difficile* metabolism and the innate T cell-mediated immunity in humans with hypervirulent strains being most competent in provoking mucosal-associated invariant T (MAIT) cell activation. A report by Horvat and Rupnik established an *in vitro* model for co-cultivation and described how different fecal microbiota samples have an impact on *C. difficile* growth and sporulation as well as the microbiota itself.

Suitable animal models are widely discussed in the scientific community. Accepted model systems are hamster and mouse models, both with specific limitations and drawbacks. Nevertheless, the murine model systems contribute valuable information on the *in vivo* role of bacterial proteins and probiotics. Combining a murine model system with enzymatic assays, Ünal et al. identified the role of the peptidyl-prolyl-cis/trans-isomerase PrsA2 as a virulence modulator influencing crucial processes such as sporulation, germination and bile acid resistance. Quigley et al. identified a *Lactobacillus grasseri* APC 678 in a co-culture system as well as in a mouse model as potential probiotic to target CDI by increasing the bacterial diversity. In a study by Xu et al., it was shown that *Pediococcus pentosaceus* exhibited a protective effect by improving the functionality of tight junctions resulting in down-regulation of the inflammatory response. In a targeted approach, Peng et al. showed that a fusion protein of bacteriophage lysin with human defensin effectively mitigated symptoms of CDI and reduced spore and toxin levels in the murine model.

## Metabolism

The unique metabolism of *C. difficile* employs multiple fermentation pathways coupled to membrane ion gradient formation for ATP generation. Major pathways utilize branched chain amino acids, cysteine, proline and further amino acids with the formation of corresponding organic acids. In parallel, the central carbon metabolism comprises a versatile sugar catabolism and an incomplete tricarboxylic acid cycle to prevent unnecessary NADH formation. Neumann-Schaal et al. summarized central features of the metabolism with a focus on the metabolic principles of energy generation. Changes of the metabolism during the onset of stationary phase were the focus of the publication by Hofmann et al. showing complex and highly specific adaption to substrate availability. In a multi-omics approach, Berges et al. provided a detailed picture on iron limitation and regulation even beyond the *fur* regulon in *C. difficile*. The important role of bile acids on germination and sporulation are widely discussed and Sievers et al. draw our attention to the stress response of *C. difficile* upon bile acid exposure. In a study on ribotype 027 isolates, Steglich et al. reported on the loss of an ABC transporter and the impact on the metabolism.

## Conclusions and Outlook

The current special issue “The Deadly Secrets of *C. difficile* – Insights into Host-Pathogen Interaction” with almost 30 contributions to toxin function, epidemiology, physiology, gene regulation and metabolism provides broad novel insights into the molecular strategies of a deadly bacterial pathogen. Since corresponding detrimental infection are still highly relevant, especially to our more and more elderly societies, these multiple novel insights into certain molecular aspect of the infection process might provide a nucleus for further therapeutic strategies. Future investigation might include holistic system biology approaches for the dynamic integration of the knowledge on the various detailly worked out adaptation strategies. Bioinformatic-based modeling and predictions about the *C. difficile* infection process might constitute a solid knowledge basis for the development of novel intervention strategies.

## Author Contributions

All authors listed have made a substantial, direct, and intellectual contribution to the work and approved it for publication.

## Conflict of Interest

The authors declare that the research was conducted in the absence of any commercial or financial relationships that could be construed as a potential conflict of interest.

## Publisher's Note

All claims expressed in this article are solely those of the authors and do not necessarily represent those of their affiliated organizations, or those of the publisher, the editors and the reviewers. Any product that may be evaluated in this article, or claim that may be made by its manufacturer, is not guaranteed or endorsed by the publisher.

## Footnotes

^1^ https://www.cdc.gov/cdiff/index.html
